# Inhibition of Replication Fork Formation and Progression: Targeting the Replication Initiation and Primosomal Proteins

**DOI:** 10.3390/ijms24108802

**Published:** 2023-05-15

**Authors:** Holly M. Radford, Casey J. Toft, Alanna E. Sorenson, Patrick M. Schaeffer

**Affiliations:** Molecular and Cell Biology, College of Public Health, Medical and Veterinary Sciences, James Cook University, Douglas, QLD 4811, Australia

**Keywords:** DNA replication, antibiotics, primase, helicase, high-throughput screening, replication fork, replisome, DnaA, bacteria

## Abstract

Over 1.2 million deaths are attributed to multi-drug-resistant (MDR) bacteria each year. Persistence of MDR bacteria is primarily due to the molecular mechanisms that permit fast replication and rapid evolution. As many pathogens continue to build resistance genes, current antibiotic treatments are being rendered useless and the pool of reliable treatments for many MDR-associated diseases is thus shrinking at an alarming rate. In the development of novel antibiotics, DNA replication is still a largely underexplored target. This review summarises critical literature and synthesises our current understanding of DNA replication initiation in bacteria with a particular focus on the utility and applicability of essential initiation proteins as emerging drug targets. A critical evaluation of the specific methods available to examine and screen the most promising replication initiation proteins is provided.

## 1. DNA Replication: A Valid Antibacterial Drug Target

**Figure 1:**
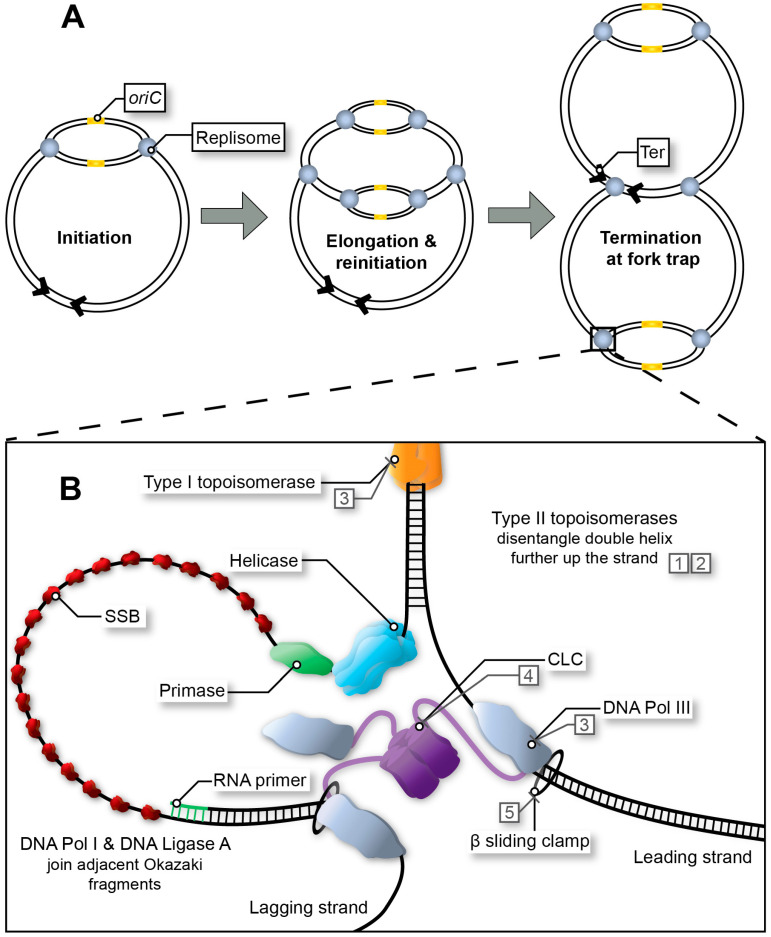
Replication fork progression from initiation to termination. (**A**) Multi-fork replication in fast growing bacteria. Multiple replisomes may load sequentially at the origin site (initiation at *oriC* in *E. coli*), as a result of reduced generation time. (**B**) Bacterial replisome during fork progression (elongation stage). As the helicase unwinds the replication bubble, RNA primers are synthesised for extension by DNA Pol III*. Polymerase activity is mediated by the β sliding clamp and its clamp loading complex (CLC). Exposed single-stranded DNA (ssDNA depicted here using a single line) is protected by single-stranded binding protein (SSB) throughout this process. In *E. coli*, adjacent Okazaki fragments on the lagging strand are joined by DNA Pol I and DNA Ligase A (not depicted). Topoisomerases release tension in the double helix as the fork progresses. Type I topoisomerases achieve this by nicking one strand within the double helix, whereas type II topoisomerases disentangle double-stranded DNA (dsDNA) by cutting both strands and passing one duplex DNA through the other to remove loops created by supercoiling. Examples of inhibitors indicated by numbered boxes (1–5) are described in Table 2.

In *Escherichia coli*, two large multimeric protein complexes, referred to as replisomes, assemble at a single, unique replication origin (*oriC*) of the circular chromosome. Each replisome proceeds in opposite direction at approximately 60 kb/min, duplicating the leading and lagging strand until a terminus site is reached (Figure 1A) [1,2,3,4]. In favourable conditions, bacterial DNA replication can occur in an overlapping manner termed multi-fork replication, whereby a second round of replication can begin prior to the first-round finishing (Figure 1A) [5,6]. Multi-fork replication is required for rapid growth of *E. coli* with generation times as low as ~20 min and the inheritance of partially replicated chromosomes in daughter cells. Regardless, DNA replication must be completed faithfully to guarantee the integrity of the genome. Whilst bacterial chromosome replication is often described based on the well-characterised *E. coli* system, significant variations do exist in other bacteria (Table 1) [1,3,4,7,8,9,10,11].

The replication process can be classically grouped into three main stages: initiation, elongation, and termination. Irreversible and unresolved stalling of fork progression by an inhibitor in any of these stages would be lethal for replicating bacteria. As such, the molecular mechanisms and essential proteins involved in chromosome replication are attractive drug targets for the development of new antibiotics, a paradigm that has been thoroughly recognised and reviewed over the past decade [9,12,13,14,15]. However, only one class of approved antibiotics is currently targeting bacterial DNA replication by inhibiting fork progression (Figure 1B) (Table 2). During elongation, topoisomerases release tension on the double helix by nicking (type I topoisomerase) and disentangling (type II topoisomerase) the strands ahead of the replication fork (Figure 1B). Quinolones and their fluoroquinolone derivatives selectively inhibit type II topoisomerases, such as the gyrase, and have been in clinical use since the 1960s. Now, increasing reports of severe side-effects have led to revocation of some quinolone derived drugs [16]. Adverse reactions included but were not limited to both hypo- and hyperglycemia, tendinitis, and even cardiac complications. Mutations within the catalytic site of type II topoisomerases have also resulted in quinolone-resistant strains, and whilst there have been some developments in non-quinolone-based antibiotics targeting topoisomerases [17], the need for the characterisation of additional replication proteins for use in drug development is of the utmost importance (Figure 1B) [18].

The *E. coli* DNA Pol III core, clamp loading complex (CLC), ß sliding clamp, DNA Pol I, DNA Ligase A, helicase, primase, and their homologs in other species (Table 1) are all involved in the essential DNA synthesis step [19] (Figure 1B). In *E. coli*, the replisome has been shown to be kinetically discontinuous [3,20]. As such, the leading-strand and lagging-strand polymerases could function independently. Graham et al. suggest that the stochastic progression of individual replisomes can replicate the chromosome without the need for coordination of synthesis of both strands [20]. The potential of most of these replisomal proteins and the multitude of interactions they form as attractive drug targets has been previously evaluated and reviewed [9,14].

**Figure 2:**
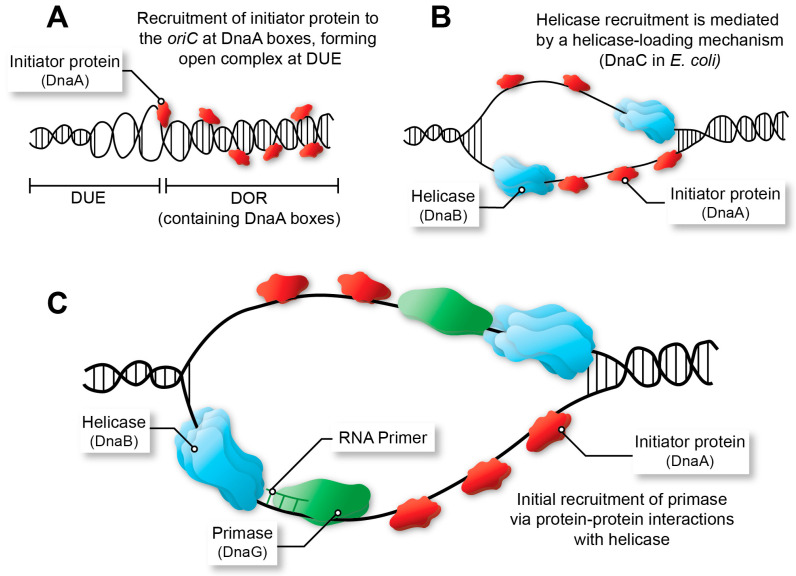
Initiation of DNA replication and the formation of the primosome at the origin (*oriC* in *E. coli*). (**A**) The initiator protein (DnaA) instigates chromosome replication through its binding to specific motifs within the DnaA-oligomerisation region (DOR), melting the DNA at the DNA unwinding element (DUE) to form the open complex. (**B**) Two hexameric helicases (DnaB_6_) as well as SSB tetramers (not depicted) are recruited to expand the replication bubble. (**C**) Final stage of initiation involving recruitment of the primase (DnaG) and initial RNA primer synthesis for the leading strand.

While the final DNA replication termination step could seem a great target, the molecular mechanisms used for coordinated replication termination are mostly non-essential, even in bacteria that use well-characterised replication fork traps [21], and are known to differ greatly even among bacteria within a single order (Figure 1A) [2,22,23,24]. As such, they are not considered of interest as drug targets and are not discussed in any further detail. This review focuses on the conserved proteins and interactions required in replication initiation and also in the primosome, specifically the initiator protein (DnaA), helicase (DnaB) and primase (DnaG). Mechanistic aspects of initiation and priming are detailed (Figure 2), with an emphasis on the characteristics of initiation and primosome proteins making them safe and specific targets for antibacterial drug development. The various mechanisms and sequential recruitment of the initiator, helicase, and primase proteins are discussed with specific attention given to alternative mechanisms of helicase loading [25,26,27,28,29]. A systematic review of initiator, helicase and primase inhibitors is provided. Finally, promising methods are evaluated with a focus on their advantages and limitations in screening and characterising inhibitors in future drug discovery campaigns.

## 2. Targeting DNA Replication Initiation 

The initiator protein, helicase, and primase are recruited to the origin of replication (*oriC* in *E. coli*) to create the initial RNA primers that will be extended (leading strand) by the DNA polymerase after replisome assembly (Figure 2). All three are essential for maintaining the stability of the genome and for the formation of new, viable bacterial cells, and thus have potential to be exploited as targets in drug discovery campaigns [13,15,30]. The helicase and primase are the most important proteins in the primosome that synthesises RNA primers at regular intervals on the lagging strand and for DNA replication restart [31]. While single-stranded DNA binding protein (SSB) and DnaB are sufficient to recruit DnaG [32] to a functional replication fork, other proteins are sometimes necessary when a replication fork has stalled and is ‘abandoned’ [33]. In *E. coli* this requires a set of DNA replication restart proteins, PriA, PriB, PriC, DnaT along with SSB to reassemble the helicase and primase [31]. This particular system is commonly referred to as the replication restart primosome. Of note, only PriA is conserved across all bacteria. In *Mycobacteria*, no homolog for any other replication restart protein has been identified [9]. While PriA is essential and kaempferol has been identified as a putative inhibitor of the *Staphylococcus aureus* protein [34], no further compound showing activity on PriA or other replication restart proteins has been reported and as such will not be further discussed.

The bacterial replication initiator, helicase and primase have functional analogs in eukaryotes but share very little sequence homology [35,36]. Thus, they can be safe and specific targets of inhibitors within a mammalian host. Comparatively, the protein sequences of the initiator, helicase and primase are relatively well-conserved amongst bacteria. Of note, the primase shows the greatest divergence in sequence conservation between Gram-negative and Gram-positive bacteria, suggesting its potential as a narrow-spectrum target [37]. The same degree of sequence conservation is not usually observed for other initiation proteins. For example, helicase loading onto single-stranded DNA (ssDNA) can be mediated by different proteins [38], undermining the potential of the helicase-loader proteins as drug targets.

The druggability of the initiator, helicase and primase is evidenced by their capacity to bind and be inhibited by small, drug-like molecules. For example, both DNA and nucleotide triphosphate (NTP) interactions by the *Mycobacterium tuberculosis* primase were inhibited by competitive binding of suramin and doxorubicin [39]. Flavonols have been shown to inhibit the *Klebsiella pneumoniae* helicase by competitively binding to its ATP-binding pocket, a similar mechanism to which has been suggested to inhibit the initiator of *E. coli* by bisindoles [40,41]. NMR screening assays have identified a novel ligand-binding site on the *S. aureus* primase separate from those previously characterised as cofactor-binding sites, showing potential for non-competitive inhibition of the protein [42]. Overall, these proof-of-principle data establish the initiator, helicase and primase as specific and druggable targets [43].

### 2.1. The Initiator Protein

The initiator protein (DnaA) promotes the formation of an “open complex” with the help of auxiliary proteins, “melting” the DNA double helix at the *oriC* (Figure 2A) [44,45,46]. This action prompts the recruitment of the primosome to the origin site which launches replication initiation and elongation. Therefore, DnaA has significant potential as a target in prospective drug discovery studies. The mechanisms of DnaA in replication initiation have been of interest in the field since the 1990s and have been extensively reviewed [47,48,49,50,51].

In *E. coli*, the cellular abundance of DnaA (both active and inactive forms) varies from 500 up to 2000 monomers depending on the growth phase of the cell [52,53]. The position of the highly conserved *dnaA* gene in proximity to the *oriC* allows for tight regulation of DnaA abundance. High concentrations of DnaA in this area, i.e., during replication initiation, downregulate transcription and translation of new DnaA protein [54]. DnaA proteins interact with DNA by binding specific, highly conserved sequences throughout the DnaA-oligomerisation region (DOR) of the *oriC*. The 9-mer (TT^A^/_T_TNCACA) is considered the perfect *E. coli* DnaA box and occurs three times in this region [47,48,55]. A further nine, closely related sequences (up to three mismatches) also appear in the DOR. Thus, a high density of these boxes often indicates the location of the replication origin and is used, along with *dnaA* gene position and GC skew, to locate putative origin sites [56].

DnaA monomers interact transiently with DnaA boxes through their C-terminal domains (domain IV) [57]. The binding of ATP and ADP to the AAA+ domain (domain III) stabilises this protein-DNA interaction. Domain III of DnaA contains typical Walker A/B, Sensor I/II, and Arg-finger motifs for ATP recognition [48,58]. Whilst either ATP or ADP-bound forms of DnaA are capable of initiation, the increased affinity of individual DnaA boxes for ATP- over ADP-bound forms allows for tighter regulation of replication [59]. A conformation change occurring when DnaA binds ATP is thought to depend on the His136 residue which is also essential for helicase recruitment. After this conformation change, DnaA oligomerisation occurs in a head-to-tail fashion between the Arg285 finger of domain III and the bound ATP of the adjacent DnaA monomer [60]. Up to 20 DnaA monomers can organise into a superhelical form, prompting the cooperative binding of ATP-DnaA to sequential DnaA boxes [47]. As this sequential binding is what ultimately leads to the melting of the DNA unwinding element (DUE) to form the open complex, ATP-DnaA is therefore considered to be the active form of the protein [1,55,61].

The interaction between DnaA and the helicase–helicase loader complex is of utmost importance [60]. Here, the complex protein–protein interactions (PPI) between DnaA and the helicase–helicase loader complex assist in loading the helicase at the correct position in the open complex of the *E. coli* chromosome.

### 2.2. Other Factors Associated with the Origin and Initiation

Newly formed DnaA is thought to bind preferentially to ATP [57,62]. In the exponential growth phase, Hda and *datA* assist with ATP-DnaA to ADP-DnaA conversion to avoid hyperinitiation [63,64]. Additional proteins, HU, IHF, Fis and DiaA, further support DnaA in the initiation stage, encouraging and stabilising oligomerisation by interacting with DnaA directly or bending the DNA into an advantageous formation for DnaA binding [51,65,66]. Of note, IHF binds to a specific sequence that overlaps one of the DnaA boxes in the DOR, as well as to the chromosomal *datA* locus. Not only does this binding sequester DnaA boxes, but it also prompts the DatA-ATP hydrolysis system as initiation of replication occurs [64,67]. Before a new cycle of replication is initiated, nucleotide exchange from ADP-DnaA to ATP-DnaA occurs at two chromosomal DnaA-reactivating sequences (*DARS1* and *DARS2*), each containing three DnaA boxes [68]. The *DARS2* locus is stimulated by Fis and IHF interactions, acting synergistically with *DARS1* to regenerate ADP-DnaA into its active form [69]. Interactions between DnaA and other replisome and accessory proteins are mediated by the N-terminal domain [70]. For example, through this domain three DnaA monomers interact with a single DiaA monomer to encourage their oligomerisation through increased proximity [48]. Two DnaA monomers are also able to dimerise through interactions between their N-terminal domains, which may promote oligomerisation, or the interactions needed for nucleotide exchange as discussed earlier [71].

### 2.3. The Helicase

DnaB is the protein subunit that forms the functional hexameric helicase in *E. coli*. The helicase follows the action of DnaA, unwinding and expanding the open complex to form a replication bubble (Figure 2B) [72]. To achieve this, a toroidally shaped helicase is recruited to encircle each ssDNA strand (Figure 2B). The helicase then translocates from 5′ to 3′, with the ssDNA passing through the central cavity. This action pushes the replication fork and forces the DNA double helix to unwind. Inhibition of the helicase would result in irreversible stalling of the replication fork. For this reason, the replicative helicase is an appealing target for new antibacterial therapeutics. Typically, approximately 120 DnaB corresponding to 20 helicases are present during both stationary and exponential growth phases, suggesting regulation is not as highly associated with protein abundance as is seen with DnaA [73]. Magnesium ions are needed to form and stabilise the hexameric structure [74]. Binding occurs at the C-terminal domain of the protein triggering the DnaB-DnaB interactions for oligomerisation. Several PPI occur during initiation, in the primosome as well as in the replication restart primosome [1,31], primarily including the initiator, helicase loader and the primase [60,75,76,77,78].

The N-terminal domain of DnaB is responsible for the interaction of the helicase with several proteins [1]. As discussed above, DnaA interacts with the helicase to ensure it assembles at the correct position in the DUE. The helicase also interacts with ATP and a loading protein in order to encircle ssDNA in the right direction. In *E. coli*, this helicase-loading protein is DnaC. Each DnaB subunit is tightly bound by DnaC in the helicase–helicase loader complex [79,80]. Importantly, DnaC traps the helicase ring into an open ATP-bound conformation, allowing its assembly around ssDNA. The DnaB-DnaC interactions also prevent binding with the primase until loading has occurred [75,79]. DnaC itself contains an ATP domain, which is thought to assist in the opening and closing of the helicase ring [1]. Following the delivery of the helicase–helicase loader complex at the DnaA-bound *oriC*, DnaC proteins dissociate. However, no direct interaction between DnaA and DnaC has been characterised. Of note, the mechanism of helicase loading by DnaC is not conserved amongst bacteria (discussed in Section 2.5), and due to this, undermines its potential as a target for drug development. Other helicases, such as UvrD involved in DNA damage repair, have been extensively reviewed [81,82]. These helicases will not be discussed further in this review.

After the helicase loader dissociates, the helicases can translocate in a 5′-3′ direction with concurrent ATP hydrolysis opening the replication bubble [83] and interact with a primase. A transient interaction between the two proteins occurs between the linker that joins the N- and C-terminal domains of DnaB and the C-terminal helicase binding domain (HBD) of the primase resulting in domain swapping [78,84,85]. The primase–helicase interaction has been shown to increase the DNA binding activity of both proteins in *E. coli* [79]. Interestingly, in *Clostridium difficile* the helicase itself is not active until association with the primase has occurred [86], while the T7 bacteriophage helicase is genetically fused to the primase [87], possibly indicating an alternative role in helicase loading.

### 2.4. The Primase

The monomeric bacterial primase is an essential protein that synthesises RNA primers for DNA replication (Figure 2C) and is an attractive target for antibiotic development campaigns [37,88]. Recruitment of the primase to the primosome completes replication initiation, activating helicase translocation and the elongation stage of DNA replication. Each helicase typically interacts with two to three primase monomers, that are present at a cellular abundance up to 100 primases throughout the growth phase [73,89,90]. RNA primers vary between 9 and 14 nucleotides in length, at intervals of approximately 1.5 kb, to form the basis for the Okazaki fragments in the elongation phase. However, both the length and frequency of these primers are known to be heavily influenced by the strength of the interactions with the helicase [91].

The mechanism of the primase relies on the action of three, distinct functional domains all of which have been extensively characterised. The C-terminal HBD interacts with the helicase [78,84]. This interaction has been shown to reduce the processivity of the replisome during replication [92]. The primase is also known to mediate the switch between initiation and elongation by inducing a conformation switch in the helicase [93]. The change causes the helicase to increase its affinity for the clamp loading complex in the DNA Pol III* holoenzyme. Thus, an increased primase concentration (within physiological boundaries) results in an increase in Pol III* residing at or near the replication fork. The primase is dependent on interactions with DNA for its polymerase activity. The N-terminal zinc-binding domain (ZBD), featuring a zinc ribbon motif ensures that the correct protein conformation is maintained for DNA binding activity [94,95]. The central RNA polymerase domain (RPD) responsible for RNA primer synthesis is highly conserved in both bacteria and bacteriophages. Crystal structures of the *M. tuberculosis* RPD complexed with DNA revealed the subdomains that interact with the DNA for primer synthesis and provides the basis for a model of primer elongation [96].

DnaG interacts with SSB. SSB has a cellular abundance of up to 2000 tetramers, coating ssDNA to prevent nucleolytic attack and the formation of secondary structures that would impede the replisome [73,97] (Figure 1B). The many roles SSB plays in replication have been reviewed in detail [98]. Not only does DnaG interaction with SSB ensure binding of the primase to the priming site, SSB interactions also allow for the hand-off of the DNA primer from the primase to the CLC and DNA polymerase for processing [99]. From here, the initiation phase of replication is complete, and the elongation phase begins. While SSB is essential, its relative high abundance, tight binding to ssDNA, and small size are problematic in drug discovery. Thus, SSB will not be discussed further within this review.

### 2.5. Alternative Helicase Loading

The helicase-loading mechanism is not strictly conserved [38]. *Bacillus subtilis* possess a functional analogue named DnaI (where the helicase itself is named DnaC) [100]. Similar to *Ec*DnaC, *Bs*DnaI forms a hexameric ring with the helicase. *Bs*DnaI is larger and contains an important zinc-binding fold in its N-terminal domain that is needed for helicase binding and loading [101,102]. Though not essential for loading, the process often includes another replicative protein, here named *Bs*DnaB, to assist in efficient loading of the *Bs*DnaC-DnaI complex [100,101].

An ancestral loading protein, DciA, is also employed to load the helicase onto the DUE in some species lacking DnaC and DnaI. In *Pseudomonas aeruginosa*, DciA has been shown to specifically bind to the replicative helicase [103]. Whilst the mechanism remains to be elucidated, the protein is essential for survival as deletion of the *dciA* gene yielded no viable colonies [103]. The identification of a PPI domain in the *M. tuberculosis* DciA homolog (Rv0004) that structurally resembles DnaA strongly implies interactions between the initiator protein and the ancestral loader during replication initiation [104]. In *Caulobacter crescentus*, DciA was found to be important for fork progression as interactions between DciA and DnaB prompted conformation changes in the helicase [105,106]. Phylogenetic studies have revealed that the rise in *dnaC* and *dnaI* replaced *dciA*, suggesting that the latter is the ancestral helicase loader [103,107].

Hexameric helicase loading by the ancestral DciA, or homologous loaders such as DnaC and DnaI, still does not encompass all known helicase-loading mechanisms. A specific helicase loader in *Helicobacter pylori* is yet to be confirmed, and loading via the putatively identified HP0897 protein is yet to be recorded in vitro [108]. Instead, a self-loading mechanism is thought to be achieved by the formation of a dodecameric helicase [109]. In most species, two hexameric helicase rings are loaded into the open complex at the beginning of replication; with one ring surrounding each of the strands, they move in opposing directions to unwind the replication bubble [1]. In *H. pylori*, these two hexameric rings complex together into a head-to-head, dodecameric form much like the archaeal and eukaryotic helicases, MCM and MCM2-7 [110]. The exact loading mechanism, though known to be mediated by DnaA, is still largely unresolved [111]. In a similar way to the *E. coli* primase dissociating DnaC from the helicase, the interaction of the helicase complex with the primase in *H. pylori* dissolves the double-ring complex into two hexameric rings encircling each strand in the DUE. After dissociation, the individual hexamers are activated to begin *oriC* unwinding [111]. As the interest of this review lies in the potential of the primosome to be exploited by drug discovery campaigns, DnaC and the other helicase loaders will not be discussed further.

## 3. Replication Initiation Inhibitors

The global burden of MDR bacteria is continually increasing [18]. DNA replication offers a multitude of safe and specific drug targets, yet very few have progressed beyond validation studies (Table 2). The initiator protein, helicase and primase have been proposed to be attractive targets for drug development; however, development of inhibitors to even a pre-clinical stage is yet to be seen. To date, only preliminary screening studies have been performed to uncover feasible inhibitors targeting the initiation and primosome proteins (Table 3). The details of these inhibitors have been summarised below.

### 3.1. DnaA Inhibitors

Studies validating DnaA as a drug target have thus far been limited to *E. coli* (Table 3) [46]. Derivatives of the bisindole 3-acetoxy-2,2’-bi-1*H*-indol were examined for their ability to inhibit DnaA through a filter binding assay [41]. Of the five derivatives examined, those containing longer alkyl chains produced greater inhibitory effects, with the most potent being 3-[N-(11-carboxyundecyl)] carbamoylmethoxy-2,2-bi-1*H*-indol with an IC_50_ of 7 µM. Inhibition was outcompeted by preincubating DnaA with ATP [41]. The mechanism of action was not confirmed. Indeed, such compounds may interact with ATP or DNA posing a challenge for pathogen-over-host selectivity. The compound showed high water solubility, making it an attractive pharmacophore. Further research would be needed to confirm a direct interaction between the bisindoles and DnaA. Nucleotide analogs could be an interesting class of compounds to evaluate with DnaA. However, interference between the human purinome and these compounds is highly likely [130] and should be avoided.

### 3.2. Helicase Inhibitors

A number of studies have validated DnaB as a promising drug target (Table 3). Flavonoids, including myricetin and galangin, were found to inhibit DnaB activity in *E. coli* and *K. pneumoniae*, two of the ESKAPEE pathogens (*Enterococcus faecium*, *S. aureus*, *K. pneumoniae*, *Acinetobacter baumannii*, *P. aeruginosa*, *Enterobacter* spp. and *E. coli*) [113,114]. Myricetin was found to selectively inhibit the helicase of *E. coli* [113]. Inhibition was linked to the ATPase activity of the helicase with the apparent K_M_ for ATP decreasing significantly in the presence of myricetin. The mechanism of inhibition was proposed to be non-competitive with an IC_50_ of 11.3 ± 1.6 μM at saturating ATP concentrations [113]. Four different flavonols (myricetin, galangin, kaempferol, and quercetin) have been linked to the inhibition of *K. pneumoniae* DnaB using fluorescence quenching assays [40]. Though results showed stronger binding to myricetin, galangin was found to better inhibit ATP hydrolysis. Taken together with the fact that myricetin has been found to inhibit both T7 gp4 and another helicase homolog (RSF1010 RepA) [131] these data provide a strong case to further search flavonoids as DnaB inhibitors.

High-throughput screening of a total 186,000 small synthetic molecules unearthed five chemotypes of coumarin-based molecules as inhibitors of *Bacillus anthracis* and *S. aureus* DnaB [115]. Further characterisation of these inhibitors showed the most potent derivative (3-(7-(biphenyl-4-ylmethoxy)-4,8-dimethyl-2-oxo-2*H*-chromen-3-yl)propanoic acid) yielding MIC and IC_50_ values in the low μM range for *B. anthracis* and *S. aureus*. A non-competitive mode of action was proposed specifically for inhibiting double-stranded DNA (dsDNA) unwinding.

McKay et al. screened 230,000 commonly available compounds and found a triaminotriazine compound with low μM inhibition of DNA duplex unwinding [116]. Fifteen different derivatives of this initial compound were compared for potency, and three showed an IC_50_ of 5 μM for *E. coli*, *S. aureus* and *P. aeruginosa* replicative helicases with MIC values in the low μg/mL range. These studies validate the helicase as a promising drug target.

### 3.3. Primase Inhibitors

The *E. coli* primase has been the focus of a number of studies establishing the protein’s significance as a potential drug target (Table 3). Chu et al. screened extracts from a fermentation broth of *Penicillium verrucosum* using an *E. coli* helicase–primase activity assay. A primase inhibitor (Sch 642305) was identified and characterised as a novel bicyclic 10-membered macrolide [128]. The molecule with a modest EC_50_ showed antibacterial activity with an MIC of 40 μg/mL against an *E. coli* strain with a defective lipopolysaccharide layer and a disrupted acrAB efflux pump. Now that synthesis of the compound has been streamlined [127,129], further development may bring the inhibitor closer to clinical trials; however, to date, the mode of interaction with the bacterial primase complex remains to be determined.

Primase inhibitor screening using a scintillation proximity assay (SPA) has also identified derivatives from *Polygonum cuspidatum* that inhibit the *E. coli* DnaG [126]. Hegde et al. screened several semi-purified fractions of aqueous methanolic plant extracts [126]. An extract was found to be inhibitory, which led to a bioassay-guided fractionation of the extract. Two phenolic monosaccharides were identified as the major inhibitors with IC_50_ values of 4 and 5 μM. The inhibitors were hypothesised to interrupt the primase’s interaction with ssDNA; however, the exact mechanism is yet to be confirmed.

In silico screening of the *E. coli* primase using an extensive library of 500,000 compounds identified 79 compounds that bound to three putative “druggable” binding sites [118]. The identified compounds were then examined for their ability to disrupt primase activity using a SPA, highlighting four potential primase inhibitors all mapping back to the same druggable site. A pharmacophore was modelled and used for further screening of primase and bacterial growth inhibitors. From a selected library of 2846 compounds, eight inhibitors were identified with IC_50_ values < 100 μM, three of which also inhibited bacterial growth in vitro. Of these, a number of derivatives were investigated with the most potent being a benzo[*d*]pyrimido[5,4-*b*]furan derivative with an IC_50_ of 1.6 μM and an MIC of 4 μg/mL. Whilst a direct interaction between the inhibitors and the primase is yet to be confirmed the data are promising for future drug development.

A pyrophosphatase based high-throughput screening was developed and applied for *M. tuberculosis*, *B. anthracis* and *S. aureus* primase activity [39,119,125]. A library consisting of 2556 small molecules (including food and drug administration-approved drugs, molecules that have been used in human therapy, and kinase inhibitors) was screened. Filtering the hits identified suramin, as well as doxorubicin and tilorone, for inhibition of the priming activity of *B. anthracis* and *M. tuberculosis*. Initially, suramin and doxorubicin (IC_50_ values of ~6 μM and ~8 μM, respectively) were highlighted as potent inhibitors of the primase from the H37Rv strain of *M. tuberculosis*. Due to the success of this assay, Biswas et al. further examined the same inhibitor library for the primase from the 34F2 Sterne strain of *B. anthracis* [119]. Similar to the previous screen, doxorubicin was identified as an inhibitor of *B. anthracis* DnaG (IC_50_ = 4 μM), along with tilorone (IC_50_ = 7 μM). The potency of tilorone is 10-fold higher in *B. anthracis* than *M. tuberculosis*, suggesting a degree of species selectivity. Whilst the mechanism of doxorubicin remains to be elucidated, suramin and tilorone were hypothesised to compete for the substrate (ssDNA and NTPs) binding sites of DnaG. Of note, the same assay identified dequalinium analogues to inhibit *S. aureus* primases. However, mechanistic studies showed these inhibitors are ssDNA bisintercalators [125]. *M. tuberculosis* primase was further screened yielding anthracyclines and aloe-emodin as inhibitors [120,121]. However, as is the case with many of the above validation studies, confirmation of a direct protein-inhibitor interaction is needed.

Of note, *para*-phenyl substituted tetrazoles have been identified to bind the C-terminal domain of the primase, preventing its interaction with SSB [117]. A total of 1140 fragments were screened using saturation-transfer difference nuclear magnetic resonance (STD-NMR), and after filtering of positive hits, ^15^N–^1^H heteronuclear single quantum coherence (HSQC) spectra confirmed protein-inhibitor interaction within the SSB-DnaG PPI site. More recently, Singh et al. applied a fragment-based NMR approach to screen the *M. tuberculosis* primase and characterised several compounds with μM activity [88,122,123].

### 3.4. Synopsis

These studies have unearthed a number of different inhibitors targeting replication mechanisms in bacteria. Whilst the data are promising and serve as a proof-of-concept for targeting replication initiation and the primosome, no progress to even preclinical stage has been seen for molecules inhibiting the activities of DnaA, DnaB or DnaG. This is in part due to the difficulty in determining the mechanisms of action of replication inhibitors using the methods that were available at the time. Recent progress within the field has seen the development of high-throughput assays able to overcome this limitation which are discussed in the following section.

## 4. Advantages and Limitations of Current Methods

The mechanisms of DNA replication initiation and associated PPI discussed above have potential as targets for the development of safe and effective antibiotics. Though this has been repeatedly recognised over the past 10 years [9,14], limited progress has been made towards clinical trials for hits targeting bacterial initiator, helicase, and primase proteins [30]. Largely, this is due to the formal characterisation of these initiation and primosome proteins historically being limited to the model bacteria, *E. coli* and *B. subtilis*, and some ESKAPEE pathogens. Indeed, several essential DNA replication proteins and PPIs have been found to differ significantly between the *E. coli* model and other bacteria, limiting broad-spectrum application [3,132]. In addition, there are other compounding issues associated with screening assays targeting replication processes involving multiprotein complexes, e.g., difficulty to decipher the specific mechanism of action of a hit, and failure to demonstrate in vivo activity due to various target access barriers (Table 4).

While most in silico and in vitro screening methods fail to identify compounds that demonstrate in vivo activity, cell-based assays cannot identify a direct mechanism of action or ensure selectivity. The following section and table evaluate a selection of promising methods available to characterise the functions and interactions of the proteins that have been or can be used to screen libraries of compounds.

Many activity assays used in past drug screening initiatives for replisome proteins were performed in low throughput, such as the [α-^32^P]ATP-based filter binding assay for *E. coli* DnaA (and primosome) developed by Mizushima et al. [41] and the thermally denaturing HPLC primer synthesis assay for *E. coli* DnaG developed by Griep et al. [113]. In some cases, low-throughput activity assays could be adapted to a high-throughput format, as seen with the helicase activity assays used by Lin and Huang [40] where both the ATPase and 5′-3′ DNA helicase activity assays have been adapted to 96-well plate formats for helicases from *E. coli* [138], *B. anthracis* and *S. aureus* [115,140]. The filter binding assay developed for the *E. coli* primosome [41] is another example that could be adapted to a 96-well plate format using a cell harvester and filter plates. More recent activity assays have been developed with higher throughput formats, including the coupled colorimetric primase–pyrophosphatase assay developed by Biswas et al. for *M. tuberculosis* DnaG [39,119] and the GFP reporter-based minichromosome assay developed by Klitgaard et al. for *E. coli* DnaA [134].

Most DNA replication initiation inhibitors have yet to demonstrate activity in in vivo trials [40,119,140,141,143]. For example, inhibitors of *Bacillus stearothermophilus* helicase–primase interaction [143] identified with a reverse yeast three-hybrid assay were unable to demonstrate antibacterial activity [30]. In another example, in vitro inhibitors of *S. aureus* and *B. anthracis* helicase identified with a fluorescence resonance energy transfer (FRET)-based assay demonstrated low activity in vivo, with insufficient inhibition to obtain MIC values [140]. Furthermore, specific inhibitors of *B. anthracis* primase identified with the coupled colorimetric primase–pyrophosphatase assay also failed to show in vivo activity, due to an inability to penetrate the bacterial envelope [119]. More recent studies applying molecular docking approaches [121,136] and/or fragment-based screens [117,122] have identified inhibitors with their activity yet to demonstrate in vivo. Multi-target approaches increase the potential for identification of lead compounds [128]. Dallmann et al. [145] used high-throughput parallel multiplicative target screening approach building on a PicoGreen fluorescence-based assay for the screening of *E. coli* and *B. subtilis* replisome. However, as these include multiple proteins, identifying the mechanism of action can be challenging.

One other major issue with these assays, already touched on in the previous sections, is the possibility of non-specific DNA interaction leading to assay failure. For example, Biswas et al. [119] identified doxorubicin as an inhibitor of *B. anthracis* primase with bacteriostatic effects in vivo, yet were unable to determine its direct mechanism of action. Doxorubicin is a DNA intercalator which could explain its activity [153]. Another example of this can be seen in the use of the assay developed by Fossum et al. [133] for identifying inhibitors of DnaA in *E. coli*, instead detecting inhibitors of DNA gyrase [137]. Furthermore, deferoxamine identified using a cell-based assay with an *E. coli* ATP-DnaA-‘locked’ strain [135] failed to restrict the growth of wild type cells and was found to act via iron-chelating activity. Unfortunately, these potential complications arise in many current biochemical activity assays for replisomal proteins when DNA is essential to their operation, leading to the inability to distinguish whether inhibition is due to targeting of the enzyme or interactions with the DNA itself.

In the last two decades, large scale drug screening campaigns have shifted to using methods that can detect the physical interaction of a compound with its protein target [147,149,154]. Fragment-based approaches involving high-throughput biophysical screening techniques have become a common feature in drug discovery. High-throughput surface plasmon resonance (SPR) is now sensitive enough to be used as a primary screen [155]. Of note, the interactions of helicases and primases have been characterised by SPR validating the utility of this technique for compound screening with these proteins [78,101]. In addition, SPR can be used for subsequent validation and kinetic characterisation of the interactions. However, stability of the target can be problematic and the cost of screening is high; both of which are important aspects to consider [147,148,155]. Specialised MS techniques to screen small molecules [156,157,158] include affinity selection-mass spectrometry (AS-MS) and pulsed ultrafiltration-mass spectrometry (PUF-MS) [159,160]. They are suitable for high-throughput screening of large compound libraries or natural product extracts on protein targets [161,162]. The helicase has been examined by native MS [163,164] and as such, should be amenable to AS-MS. Differential scanning fluorimetry (DSF) [150,165], also known as Thermofluor, is probably the most-commonly used technique as a first step in the process of large compound library screening due to its technical simplicity and low cost. A derivative of this technique, DSF of GFP-tagged proteins (DSF-GTP), has been validated with several *E. coli* and *Burkholderia pseudomallei* GFP-tagged replisomal proteins such as Tus, DnaA, DnaB and DnaG [151,152,166,167]. DSF-GTP has several advantages over classic DSF in that it can be used with protein mixtures and extracts to evaluate target access [152], providing a powerful platform for future large scale drug screening campaigns.

## 5. Conclusions and Perspective

Overall, replisomal proteins are clearly attractive therapeutic targets for the development of antibiotics [9,13,14,15,30,46,88,168]. In all bacteria, DNA replication starts at a specific origin of replication where origin-binding proteins bind and locally unwind the DNA. Once recruited to the origin, the helicases unwind DNA and form the initial replication bubble. The exposed ssDNA regions are coated with SSB and DNA primases synthesise the first RNA primers. DNA polymerases and the rest of the replisome are recruited for bidirectional DNA synthesis. These processes are fundamentally conserved in bacteria, archaea and eukarya, as well as bacteriophages. However, the proteins and complexes involved are sufficiently different to be exploited as specific drug targets [169]. While not the focus of this review, aside from topoisomerases, it is worth noting that targeting the high processivity of DNA synthesis conferred by the sliding clamp is currently attracting the most interest and has been the focus of several studies and reviews [9,170,171].

In this review, we discussed how essential factors of bacterial DNA replication initiation and the primosome can serve as targets for the discovery of new generations of antibiotics and the methods available to this end. One obvious approach to halt replication is to inhibit initiation via binding of small molecules to DnaA. However, targeting DnaB and DnaG seems to be a more potent route as it also inhibits the primosome. It is clear that while nucleotide analogs could be considered as pharmacophores, they have significant disadvantages such as high-toxicity and lack of pathogen-over-host selectivity. For example, nucleoside analogs interacting with the mitochondrial polymerase γ could lead to toxicity by inhibiting mitochondrial DNA replication [172]. Drug discovery projects targeting bacterial helicases have yielded very few compounds. Nevertheless, the druggability of the human replicative helicase for anticancer drug development provides support to continue investigating this route. The advantages and limitations around the use of human helicase inhibitors have been previously discussed and summarised [173,174]. Some of these considerations are directly relevant to bacterial helicases and inhibitors. Campaigns targeting the primase have been more successful with the identification of specific macrolide inhibitors, a class of compounds that have a notoriously high pharmacological hit rate. Simultaneous targeting of these multiple replication proteins could lead to more effective treatment strategies and less resistance. In their review, Reiche et al. discuss an example of a combination *M. tuberculosis* antibiotic strategy simultaneously depleting dNTP pools while inhibiting DNA polymerase activity [9].

More structural and functional information is still needed to fully understand the replisome architecture and interactions. High resolution protein structures will allow better docking of small molecules and to examine their effect on essential replisome interactions. We anticipate that the employment of newer high-throughput assays as well as in silico methods will address some of the previous challenges and accelerate the development of novel strategies to specifically halt DNA replication and ultimately identify effective antibacterial drug treatment.

## Figures and Tables

**Table 1:** Homologs of major replication proteins between *Escherichia coli*, *Bacillus subtilis*, *Helicobacter pylori*, *Mycobacterium tuberculosis*, and bacteriophages T4 and T7.

	*E.* *coli*	*B. subtilis*	*H.* *pylori*	*M. tuberculosis*	Phage T4	Phage T7			
Initiation accessory proteins	IHF, HU, Fis, DiaA	DnaD, YabA, SirA	HobA, HU	-	-	-	Initiation		
Initiator protein	DnaA	DnaA	DnaA	DnaA	-	T7 RNAP			
Helicase loader	DnaC	DnaI, DnaB	HP0897, -	DciA	gp59	-			
Replicative helicase	DnaB_6_	DnaC_6_	DnaB_6,12_	DnaB_6_	gp41	gp4		Primosome	
Primase	DnaG	DnaG	DnaG	DnaG	gp61	gp4			
SSB ^1^	SSB_4_	SSB_4_	SSB_4_	SSB_4_	gp32	gp2.5_2_			
CLC ^2^	${\left(\tau /\gamma \right)}_{3}\delta \delta \prime \chi \psi$	$\tau_{3\;}\delta \delta \prime$	${\left(\tau /\gamma \right)}_{3}\delta \delta \prime$	$\tau_{3\;}\delta \delta \prime$	gp44_4_gp62	-	Elongation		
Sliding clamp	$\beta_{2}$	$\beta_{2}$	$\beta_{2}$	$\beta_{2}$	gp45_3_	Trx (*E. coli*)			
DNA Pol III core	αεθ	PolC, DnaE	αε	DnaE1 (αε)	gp43	gp5			
Terminator	Tus	RTP_2_	-	-	-	-	Termination		

^1^ Single-stranded DNA binding protein; ^2^ Clamp loading complex. Proteins involved in initiation (red), primosome (white), elongation (blue) and termination (green).

**Table 2:** Status of inhibitors listed in DrugBank database that target replication fork progression.

	Inhibitor	Status	Target	Access. No.
**1**	Quinolones/Fluoroquinolones (Nalidixic acid derivatives)	Clinical use	Type II topoisomerases	DB00779
**2**	Aminocoumarins (Novobiocin)	Withdrawn	Type II topoisomerase (specifically Gyrase B)	DB01051
**3**	Thymidine monophosphate	Experimental	DNA topoisomerase I DNA Pol III (ε subunit)	DB01643
**4**	Adenosine 5′-[gamma-thio]triphosphate	Experimental	CLC ^1^ (τ subunit)	DB02930
**5**	[(5R)-5-(2,3-dibromo-5-ethoxy-4-hydroxybenzyl)-4-oxo-2-thioxo-1,3-thiazolidin-3-yl]acetic acid	Experimental	Sliding clamp (β)	DB06998

^1^ Clamp loading complex. Numbers 1–5 correspond to target locations in Figure 1.

**Table 3:** Inhibitors targeting the initiator, helicase and primase proteins.

Protein Target	Inhibitor	Species *	Ref.
DnaA	Bisindole derivatives	*Eco*	[41,112]
DnaB	Myricetin	*Eco*	[113]
	Galangin	*Kpn*	[114]
	Coumarin-based	*Ban* *Sau*	[115]
	Triaminotriazine derivatives	*Eco* *Sau* *Pau*	[116]
DnaG	*Para*-phenyl substituted tetrazoles	*Eco*	[117]
	Benzo[*d*]imidazo[2,1-*b*]imidazoles Benzo[*d*]pyrimido[5,4-*b*]furans Pyrido[3’,2’:4,5]thieno[3,2-*d*]pyrimidines	*Eco*	[118]
	Tilorone Doxorubicin Suramin	*Ban*	[119]
	Doxorubicin Suramin Ellagic acid	*Mtb*	[39]
	Anthracyclines and aloe-emodin	*Mtb*	[120]
	Daunorubicin derivatives	*Mtb*	[121]
	3-[1-benzyl-5-chloro-2-(ethoxycarbonyl)-4-(trifluoromethyl)-1*H*-indol-3-yl]propanoic acid 3-[1-benzyl-7-chloro-2-(ethoxycarbonyl)-5-(trifluoromethyl)-1*H*-indol-3-yl]propanoic acid	*Mtb*	[122,123]
	9-fluorenone-based derivatives	*Sau* *Ban* *Bth*	[124]
	Dequalinium analogues	*Sau*	[125]
	Phenolic monosaccharides derived from *Polygonum cuspidatum*	*Eco*	[126]
	Bicyclic 10-membered macrolide (Sch 642305) from *P. verrucosum*	*Eco*	[127,128,129]

* *Eco*—*Escherichia coli; Kpn*—*Klebsiella pneumoniae; Ban*—*Bacillus anthracis; Sau*—*Staphlococcus aureus; Pau*—*Pseudomonas aeruginosa; Mtb*—*Mycobacterium tuberculosis; Bth*—*Burkholderia thailendensis*.

**Table 4:** Screening assays for the initiator, helicase and primase proteins.

Target	Species *	Type	Advantages	Disadvantages	Ref.
DnaA	*Eco*	Cell-based (dnaA219rnhA reporter strain)	Compounds can cross bacterial membrane	Cannot distinguish compounds with specificity for DnaA	[133]
	*Eco*	Minichromosome-based (GFP reporter)	Compounds can cross bacterial membrane	Inhibition of plasmid-based *oriC* not always repeatable with chromosomal *oriC*	[134]
	*Eco*	Cell-based (pBR322-*DARS2* and *hda* mutant reporter strains)	Compounds can cross bacterial membrane	Cannot distinguish compounds with specificity for DnaA	[135]
	*Eco*	Filter binding assay ([α-^32^P]ATP)	Could be converted for HTS	Requires radioactive labelling and scintillation counter	[41]
	*Spy*	In silico (molecular dynamics simulation)	Inexpensive, can be used for pre-screening	Requires additional in vivo efficacy conformation	[136]
	*Eco*	Cell-based (SF53 reporter strain)	HTS format (384-well plates)	Cannot distinguish compounds with specificity for DnaA	[137]
DnaB	*Kpn*	ATPase assay (molybdophosphoric acid complex)	Could be converted for HTS	Low throughput	[40]
	*Kpn*	Helicase activity assay (FRET ^1^)	Could be converted for HTS	Low throughput	[40]
	*Sau*	ATPase assay (molybdophosphoric acid complex)	HTS format (96-well plates)	Requires helicase activity assay for confirmation	[115]
	*Eco* *Sau* *Ban* *Pau*	Helicase activity assay (FRET ^1^)	HTS format (96-well plates)	Cannot distinguish compounds with specificity for DnaB	[115,116,138,139,140]
	*Kpn*	dNTP dissociation (fluorescence)	Could be converted for HTS	Requires helicase activity assay for confirmation	[114]
	viral and bacterial	Time-resolved FRET ^1^ (Tb^3+^, Eu^3+^)	HTS format (Up to 1536-well plates)	Optical interference from compounds	[141]
	*Eco* *Bst*	ATPase assay (NADH)	Could be converted for higher throughput	Low throughput	[113,132]
	*Bst*	Helicase activity (radioactive label not specified)	Can be used for inhibitors of DnaB/G interaction	Low-throughput, requires radioactive labelling	[132]
UvrD	*Eco*	Helicase activity (SYTOX stain)	Microfluidic flowcell format, could be used for DnaB	Low throughput	[142]
DnaB/ DnaG	*Bst*	Reverse yeast three-hybrid (β-galactosidase)	Allows screening of potential antimicrobial peptides	Low-throughput, for screening of peptides only	[143]
	*Eco*	SPA ^2^ ([^3^H]CTP)	HTS format (96-well plates)	Requires radioactive labelling and scintillation counter	[118,138]
DnaG	*Eco*	Thermally denaturing HPLC (260 nm)	Can distinguish between de novo synthesis and elongation	Low throughput	[113,144]
	*Mtb* *Ban* *Sau*	Pyrophosphatase assay (molybdophosphoric acid complex)	HTS format (384-well plates)	Cannot distinguish compounds with specificity for DnaG	[39,119,120,125]
	*Eco* *Mtb*	Primase activity ([^3^H]NTP, [α-^32^P]ATP)	Can be used for inhibitors of DnaB/G interaction	Low throughput, requires radioactive labelling, cannot distinguish compounds with specificity for DnaG	[123,126,128]
	*Eco*	SPR ^3^ competition assay	Can be used for inhibitors of DnaG/SSB interaction	Cannot distinguish compounds with specificity for DnaG	[117]
	*Eco* *T7*	STD ^4^ and 2D NMR (I_max_, ^15^N-^1^H HSQC ^5^)	Could be used for inhibitors of other proteins	Requires pooling of compounds for initial screens	[117,122]
	*Eco*	Primase/Replicase activity assay (PicoGreen)	Can be used for inhibitors of DnaG/SSB interaction, HTS format (384-well plates)	Cannot distinguish compounds with specificity for DnaG	[145,146]
All	*Any species*	Molecular docking (AutoDock Vina, Glide, Molecular Operating Environment)	Can give indication of mechanism of action	Requires additional in vivo efficacy conformation	[117,118,121,122,136]
	*Any species*	SPR ^3^	Sensitive, can determine binding kinetics	Expensive, need to control for buffer effects	[147,148]
	*Any species*	Mass spectrometry (AS-MS ^6^, LC-ESI-MS ^7^)	Sensitive, can pool compounds to increase throughput	Requires multiple rounds for inhibitor ranking	[147,149]
	*Any species*	Thermofluor (SYPRO Orange & ANS stain)	HTS format (384-well plates)	Optical interference from compounds	[147,150]
	*Any species*	DSF-GTP ^8^ (GFP)	HTS format (96-well plates), can be used in mixed samples, can test target access	Optical interference from compounds	[151,152]

* *Eco*—*Escherichia coli; Spy*—*Streptococcus pyogenes; Kpn*—*Klebsiella pneumoniae; Sau*—*Staphlococcus aureus; Ban*—*Bacillus anthracis; Pau*—*Pseudomonas aeruginosa; Bst*—*Bacillus stearothermophilus; Mtb*—*Mycobacterium tuberculosis; T7*—*T7 bacteriophage*. HTS:High-throughput screening. ^1^ Fluorescence resonance energy transfer; ^2^ Scintillation proximity assay; ^3^ Surface plasmon resonance; ^4^ Saturation transfer difference; ^5^ Heteronuclear single quantum coherence; ^6^ Affinity selection-mass spectrometry; ^7^ Liquid chromatography-electrospray ionisation-mass spectrometry; ^8^ Differential scanning fluorimetry of GFP-tagged proteins.

## Data Availability

Not applicable.
